# Strengthening digital health equity by balancing techno-optimism and techno-skepticism through implementation science

**DOI:** 10.1038/s41746-023-00954-0

**Published:** 2023-11-02

**Authors:** Jorge A. Rodriguez, Courtney R. Lyles

**Affiliations:** 1 Division of General Internal Medicine and Primary Care, Brigham and Women’s Hospital, Boston, MA USA; 2 Center for Healthcare Policy and Research, University of California Davis, Sacramento, CA USA; 3 Department of Public Health Sciences, University of California Davis School of Medicine, Sacramento, CA USA

**Keywords:** Health services, Patient education

## Abstract

As result of the pandemic-related increase in telehealth and the 21st Century Cures Act, technology is playing an increasing role in healthcare. This has led to organizational investments in the “digital front door” of healthcare. The promise that these technologies can revolutionize care by better connecting us to our patients, overcoming analog barriers to care, and addressing health disparities is grounded in “techno-optimism.” We advocate for organizational leaders to inform their digital health equity strategies with a balanced measure of “techno-skepticism”, grounded in implementation science, that can ensure alignment between health technology and health equity.

Over the past decade, we have expanded the use of digital tools in healthcare leading to the emergence of the “digital front door” for patients. The digital front door refers to the technologies that patients can use to engage with the healthcare system (e.g., patient portals, telemedicine, remote patient monitoring). These changes have been driven by the Meaningful Use program, the expansion of patient portals, the pandemic demonstrating the possibility of telehealth, and the 21st Century Cures Act^1–3^. The promise that technology can revolutionize care by better connecting us to our patients, overcoming analog barriers to care, and addressing health disparities is grounded in “techno-optimism.” Techno-optimism is the belief that we can use technology to address our patient’s barriers to health, and often implicit in or tangential to that belief is the idea that these technologies will spread to all populations, thereby reducing known health disparities^4^. Yet, historically marginalized communities have been left behind as healthcare has become digital, as evidenced by the recent struggle to equitably implement telehealth, especially video visits^3,5,6^. While previous work has focused on patient-level factors (e.g., influence of age, race, ethnicity, language, and digital literacy on digital health adoption and use), a renewed focus on organizational level digital health equity strategies can advance a holistic approach to widespread implementation of technology. We present the challenge of current digital health equity strategies and use implementation science to offer opportunities for the future of the digital front door.

To achieve the potential of digital tools for health equity, we need to change our implementation approach. Healthcare leaders should pursue a rigorous understanding of the problems they are trying to solve and adopt solutions that are well-matched to these problems across individual, interpersonal, system, and societal drivers. They should seek to address the disconnect between evidence-based approaches, frontline experience, and leadership decisions that promote technology use^7,8^. This process necessitates a balanced measure of “techno-skepticism”, which emphasizes questioning the use of technology for a specific problem(s). In other words, we must ask: have we balanced techno-optimism with an understanding of how technology may or may not address the multilevel drivers of health disparities? Interventions to address health disparities have increasingly focused on social and structural determinants of health, yet the digital front door strategy is not always aligned with these goals^9^. Using a techno-skeptical approach healthcare organizations can establish a comprehensive digital front door strategy that aligns with the needs of marginalized populations and thoughtfully invest in technology. It prompts healthcare leaders to ask: “How and why does the ‘front door’ of healthcare need to be digital?” and/or “What aspects of the “front door” of healthcare should remain non-digital?”

Implementation science theories, like the Diffusion of Innovation Theory or the Behavior Change Wheel, can offer the foundation for a techno-skeptical approach. The Diffusion of Innovation theory recommends considering the following concepts when implementing a new technology while ensuring alignment with the needs of marginalized communities: relative advantage, compatibility, complexity, trialability, and observability^10^. For example, while enrolling patients in patient portals may offer them the advantage of messaging their clinical teams over calling and waiting on hold, the complexity of sending and receiving messages on the portal limits access to care for some patients. The Behavioral Change Wheel, which allows an exploration of a problem from cognitive, motivational, social, and environmental/contextual domains, can guide healthcare leaders to better “diagnose” the underlying problem or behavior they seek to address and ensure the alignment of technology^11^. As an example, to promote equitable access to the digital front door, healthcare organizations may commit resources to enrolling patients in the patient portal. But enrolling in the portal is not enough to impact the drivers of healthcare disparities. These efforts should be informed by a clear identification of the problem(s) the portal will solve for patients (e.g., online medication refills or easier appointment scheduling) and what motivation(s) patients may have for enrolling (e.g., ease of sharing data with family members/caregivers or as the result of a recommendation from a healthcare provider). Being enrolled in a portal may give patients a key to the digital front door, but it does not demonstrate how digital tools play a role in their healthcare or their health.

Moving forward, healthcare leaders can take several steps to apply a techno-skeptical approach informed by implementation science to guide their organization’s digital health equity strategy and optimize the use of digital tools for health equity. (Table 1). First, understand stakeholder perspectives (e.g., patient, community, clinician, leaders) on the target health disparity and their digital readiness. For example, collaborate with community leaders and/or patient and family advisory boards to hold patient informational sessions to gauge whether a new technology will bring value to the challenges they face. Second, assess whether non-technological approaches are needed (in place of or in addition to technology) to better address the target health disparity. Third, perform early testing with end users that represent the patient/community and mimic real-world workflows. Fourth, roll out the technology that includes the relevant implementation wraparounds (i.e., training and clinical support), not just the new tool itself. Fifth, establish not only measures of digital equity (e.g., digital usage measures), but ensure health equity measures are part of the evaluation – such as process and clinical outcome measures that are stratified by core populations within the healthcare setting.

**Table 1:** Comparing approaches to technology implementation for health equity.

Vignette
As part of its organizational strategy, a healthcare organization caring for a marginalized patient population has prioritized racial/ethnic disparities in diabetes outcomes. They are tasked with developing a digital approach to address these disparities.
Techno-optimistic Approach
The healthcare leaders determine individual-level support at home for diabetes self-management should be the clear focus of the program. They move towards a technology-based approach to address these gaps that relies on a diabetes app that tracks home blood glucose values and lifestyle/behavioral domains, which can be integrated into the EHR via the patient portal. The healthcare leaders perform an environmental scan of potential diabetes platforms. They meet with app vendors and identify an app that helps patients track their blood sugar and their diet. Their key performance indicator is how many patients have downloaded and registered for the app and how clinical outcome measures (e.g., HbA1c) have changed.
Techno-skeptical Approach
The healthcare leaders engage stakeholders, including patients and communities, in refining the underlying drivers of disparities in diabetes outcomes in their healthcare system catchment area. They ask what are the core drivers of disparities in which the healthcare system directly intersects, and collectively discuss how technology plays a part in these underlying factors. Through an implementation science approach, they identify that their patients are struggling with food insecurity, particularly knowledge of the full suite of food resources in specific neighborhoods. They decide to focus on investing in social needs screening for food insecurity at the population level within their electronic health record and use technology to facilitate closed-loop referrals to community food pantries, prioritizing specific food pantry partners in under-invested neighborhoods. Their key performance indicators are how many patients have been screened and referred (including understanding the success of these new workflows within the clinics) and how clinical diabetes measures have changed (overall and stratified by patient self-reported racial/ethnic groups).

A techno-skeptical approach to the digital front door aims to ensure that healthcare technology is appropriately integrated into care and supports patients and communities to achieve health equity. The goal is not to slow the progress of innovation. Social and structural barriers drive health disparities, and despite our increasingly technology-focused society, health technology is not sufficient on its own to solve essential care gaps. A techno-skeptical approach calls for healthcare leaders to question and determine the need for the digital front door in each context to ensure these investments lead to health equity.
